# Tumor-on-chip platforms for breast cancer continuum concept modeling

**DOI:** 10.3389/fbioe.2024.1436393

**Published:** 2024-10-02

**Authors:** Anca-Narcisa Neagu, Danielle Whitham, Pathea Bruno, Nicholas Versaci, Peter Biggers, Costel C. Darie

**Affiliations:** ^1^ Laboratory of Animal Histology, Faculty of Biology, “Alexandru Ioan Cuza” University of Iași, Iasi, Romania; ^2^ Biochemistry & Proteomics Laboratories, Department of Chemistry and Biochemistry, Clarkson University, Potsdam, NY, United States

**Keywords:** breast cancer (BC), breast cancer continuum concept (BCCC), breast-cancer-on-chip (BCoC), breast cancer liquid biopsy-on-chip (BCLBoC), breast cancer metastasis-on-chip (BCMoC)

## Abstract

Our previous article entitled “Proteomics and its applications in breast cancer”, proposed a Breast Cancer Continuum Concept (BCCC), including a Breast Cancer Cell Continuum Concept as well as a Breast Cancer Proteomic Continuum Concept. Breast cancer-on-chip (BCoC), breast cancer liquid biopsy-on-chip (BCLBoC), and breast cancer metastasis-on-chip (BCMoC) models successfully recapitulate and reproduce *in vitro* the principal mechanisms and events involved in BCCC. Thus, BCoC, BCLBoC, and BCMoC platforms allow for multiple cell lines co-cultivation to reproduce BC hallmark features, recapitulating cell proliferation, cell-to-cell communication, BC cell-stromal crosstalk and stromal activation, effects of local microenvironmental conditions on BC progression, invasion/epithelial-mesenchymal transition (EMT)/migration, intravasation, dissemination through blood and lymphatic circulation, extravasation, distant tissues colonization, and immune escape of cancer cells. Moreover, tumor-on-chip platforms are used for studying the efficacy and toxicity of chemotherapeutic drugs/nano-drugs or nutraceuticals. Therefore, the aim of this review is to summarize and analyse the main bio-medical roles of on-chip platforms that can be used as powerful tools to study the metastatic cascade in BC. As future direction, integration of tumor-on-chip platforms and proteomics-based specific approaches can offer important cues about molecular profile of the metastatic cascade, alowing for novel biomarker discovery. Novel microfluidics-based platforms integrating specific proteomic landscape of human milk, urine, and saliva could be useful for early and non-invasive BC detection. Also, risk-on-chip models may improve BC risk assessment and prevention based on the identification of biomarkers of risk. Moreover, multi-organ-on-chip systems integrating patient-derived BC cells and patient-derived scaffolds have a great potential to study BC at integrative level, due to the systemic nature of BC, for personalized and precision medicine. We also emphasized the strengths and weaknesses of BCoC and BCMoC platforms.

## 1 Introduction

Organ-on-chip (OoC) platforms are engineered microphysiological biomimetic systems that reproduce the cyto-histo-architecture and physiology of human organs and tissues (Slay et al., 2021; Singh et al., 2022). As new generation of *in vitro* models, OoC systems contain natural or engineered tissues grown inside microfluidic chips that simulate cell microenvironment to maintain tissue-local conditions and functions (Leung et al., 2022; Subia et al., 2021). To investigate pathophysiology of tumors and analyse the dug efficiency in native-like environments, three-dimensional (3D) cancer/tumor-on-a-chip systems have emerged as a new generation of *in vitro* disease models that recapitulate the key hallmarks of the tumor microenvironment (TME), such as biochemical gradients, niche factors, or cell-cell and cell-matrix interactions (Imparato et al., 2022), and allow for the analysis of dynamic interactions that drive the tumor progression (Subia et al., 2021; Seaman et al., 2023). Cancer-on-a-chip models can incorporate multiple cell types and extracellular matrix (ECM) components to study the TME for multiple cancer types (Seaman et al., 2023), or to assess the side effects of anti-cancer drugs *in vitro* (Kamei et al., 2017) in different models for breast (Subia et al., 2021; Moccia and Haase, 2021), lung (Zhu et al., 2023), bone (Mansoorifar et al., 2021), glioblastoma (Xie et al., 2023) and brain (Amirifar et al., 2022), liver (Shen et al., 2023), kidney (Wang et al., 2022), heart (Kamei et al., 2017), gut (Xian et al., 2023; Zhang and Qiao, 2023), skin (Risueño et al., 2021) or lymph node and lymph node subcapsular sinus microenvironment (Shanti et al., 2021; Cho et al., 2021a; German et al., 2023; Birmingham et al., 2020). Breast cancer-on-chip (BCoC) microfluidic systems have been used to model disease progression, discover and analyze novel BC biomarkers, for drug screening applications, as well as for multiple data analysis (Subia et al., 2021).

Metastasis is the hallmark of cancer often responsible for cancer-related deaths (Fares et al., 2020). The metastatic cascade, named here Breast Cancer Continuum Concept (BCCC), emphasizes the cellular processes sustained by specific biomolecular mechanisms that initiate the transformation and progression of aggressive and invading clones of tumor cells. Moreover, breast cancer cells change their phenotype as well as their biomolecular profile, leave the primary site of the tumor, continue with the local invasion, intravasation, survival and traveling through the circulatory system, followed by extravasation and colonization of distant organs to develop metastases (Eslami-S et al., 2020). In this context to design, develop, and implement organ-on-chip platforms is absolutely necessary to highlight the molecular principles of metastasis in the context of cellular events that drive this undesirable dissemination process. Recently, Firatligil-Yildirir et al. (2023) summarized the main applications of lab-on-a-chip systems (LoC) used for BC metastasis research, in the study of invasion, migration, extravasation, modelling metabolic and biochemical proprieties, BC diagnosis, treatment and nanomedicine (Firatligil-Yildirir et al., 2023). Moreover, building risk-on-chip models based on 3D cell culture may improve BC risk assessment and prevention based on identification of biomarkers of risk (Vidi et al., 2013).

To model and decipher inter-organ communication, whole-body physiology and development of systemic diseases, multi-organ-on-chip tools coupling and integrating various tissue niches linked by the vascular flow, such as human heart, brain, lung, liver, kidney, intestine, vasculature, bone and skin tissues, have been designed and developed (Ronaldson-Bouchard et al., 2022; Picollet-D’hahan et al., 2021). Metastasis is a complex systemic disease (Alečković et al., 2019), so biomimetic microsystems able to reproduce the dynamic circulating tumor cells (CTCs) spreading to multiple organs, known as multi-organs-on-chip approach, confirm the potential for investigating multiple-organ metastasis (Kong et al., 2016). Consequently, multi-organs-on-chip systems may be used for disease modeling and monitoring of toxicity induced by cancer chemotherapy, as in case of a dual-organ platform, the heart-BC-on-a-chip (Lee et al., 2021). Zuchowska and Skorupska (2022) have reviewed a plethora of multi-organ-on-chip applications, summarizing the main applications of cancer niche-on-chip, metastases-on-chip, that includes intravasation-on-chip, CTCs detection and isolation, extravasation-on-chip, and organ specificity (Zuchowska and Skorupska, 2022).

Organ/tumor-chip models are able to recapitulate the main mechanisms of tumor development: cellular proliferation, bidirectional interaction between tumor cells and ECM components in the TME, EMT/invasion/migration, intravasation, survival in blood or lymph circulation, extravasation, distant niches construction, and immune escape of cancer cells (Li et al., 2023a). Multiple processes have been analysed by BCoC and BCMoC-based approaches, such as activation of the tumoral stroma during the neoplastic invasion (Gioiella et al., 2016), contribution of the adipose tissue on BC initiation, progression, invasion, and treatment response (Hamel et al., 2024), T cell-macrophage interaction in BC progression (Manoharan et al., 2024), blood vessel- and lymph vessel-based intravasation (Cho et al., 2021a), capture/isolation of circulating tumor cells (CTCs) from whole blood (Abdulla et al., 2022), impact of hypoxia on the extravasation of BC cells (Song et al., 2018), premetastatic niche formation within the liver by BC-derived extracellular vesicles (EVs) (Kim et al., 2020), or sympathetic modulation of BC bone metastasis (Conceição et al., 2022).

Invasion and metastasis have been considered the central hallmarks in BC progression, followed by sustaining proliferative signalling in the primary tumor, inducing angiogenesis, resisting apoptosis, enabling replicative immortality, evading growth suppressors, genomic instability, reprogramming energy metabolism, evading immune destruction, and tumor-promoting inflammation (Saha et al., 2021). Thus, tumor-on-chip models have been employed to understand metastases, multi-organ interactions, and drug efficacy and toxicity (Moccia and Haase, 2021). Microtechnology has a key role in precision bio-medicine advancement, including the use of novel microtechnology-based biosensing devices, known as smart biosensors, that contribute to a better understanding of the cancer biopathology, early disease detection and a more accurate or personalized treatment (Pereiro et al., 2021; Stella et al., 2023).

Nanobiotechnology/bionanotechnology/nanobiology, as a “miniaturized biotechnology”, is based on nanoparticles, nanodevices, and nanoscale concepts or processes that range in size from 1 to 100 nm, being also widely used in biomedical sciences (Tawade et al., 2023). There are different three-dimensional (3D) models based on different organs used in BC tumorigenesis and metastasis research, such as lymph node-on-chip (LNoC) (German et al., 2023), liver-on-a-chip (Kim et al., 2020), bone-on-a-chip (BoC) (Conceição et al., 2022; Hao et al., 2018), lung-on-chip, brain-on-chip, blood-brain barrier (BBB)-on-chip, blood-brain niche (BBN)-on-chip (Westerhof et al., 2023), brain organoid-on-chip (Cui et al., 2022), and gut microbiome-on-chip (Salehi et al., 2023). Additionally, Slay et al. (2021) showed that mechanobiology can be incorporated in next-generation OoC models, for example, for study of the bone metastasis (Slay et al., 2021).

Evidence suggests that MS-based approaches used in multiple omics-based fields, such as proteomics/peptidomics, metabolomics/lipidomics, hormonomics and others may be coupled with organ-on-chip technologies, mainly to detect the protein biomarkers from the extracellular vesicles released within microfluidic chips or to assess the effects of chemotherapeutic drugs on BC cells (Kogler et al., 2023). Thus, MS is a promising analytical tool for real-time analysis of BC cells secretomes from organ-on-chip models and new advances in the on-line connection of organ-on-chip and MS are made towards a successful hyphenation of organ-on-chip with MS, necessary to decipher the proteomic continuum concept in BC (Hadavi et al., 2023). Therefore, the aim of this review is to summarize and analyse the main bio-medical roles of on-chip platforms that can be used as powerfull tools to study the BCCC.

## 2 BCCC-on-chip

More than 90% of the related mortalities in BC are due to metastasis, known as the migration of cancer cells from the primary tumor site to distant organs or tissues (Ali Moradi et al., 2023). Metastasis cascade consists of several steps, including local proliferation of tumor clones, invasion, intravasation, circulation, extravasation, arrest at remote location, and colonization (Ali Moradi et al., 2023). Within our previous article entitled “Proteomics and its applications in breast cancer”, we proposed a Breast Cancer Continuum Concept (BCCC) that reflects the flow of the metastatic cascade, integrating multiple populations of BC cells and tumor-associated cells, which progress from the primary tumor site toward distant organotropic organs (Neagu et al., 2021). This cellular integrative flow is acompagnied by a Breast Cancer Proteomic Continuum Concept, whereas each phenotype of neoplastic cells and cancer-associated cells, within TME and distant tumoral niches, is characterized by a changing addaptive proteomic profile detected in solid or liquid biopsies (Neagu et al., 2021). Here, we give evidence for the role of BC-on-chip (BCoC), BC liquid biopsy-on-chip (BCLBoC), and BC metastasis-on-chip (BCMoC) devices to characterize the BCCC that drives the metastatic cascade at cellular and proteomic levels.

### 2.1 Breast cancer-on-chip (BCoC) platforms detect the primary tumor-related cellular and molecular events

Dissemination of BC cells precedes the initial step of the invasion in the BCCC, while the chromosomal instability promotes cancer cell to adapt to stress condition (Zheng et al., 2023). Frequenlty used BC cell lines for different tumor-on-chip devices are MDA-MB-231, MCF7, T47D, BT549, 4T1, and SK-BR-3, as well as surgically extracted patient-derived tumor cells (Li et al., 2023a), resulting in patient-derived models as promising tools in personalized medicine (Garre et al., 2022). Breast-on-chip models might mimic the tree-like ductal system of the breast (Grafton et al., 2011), including human mammary epithelial cell lines that recapitulate the epithelial cell polarity, such as MCF10A, MCF12A, 184A1, and HMT-3522S1, which have been used for study of mechanisms leading to cancer onset under the influence of different BC risk factors (Vidi et al., 2013). Toh et al. (2018) developed a 3D microfluidic cancer cell migration model to simulate the metastatic MX1 BC cells to migrate and invade across a collagen barrier that mimics the structure of the basement membrane (Toh et al., 2018). In this model, invading MX1 cells emphasized both amoeboid and mesenchymal-like motility, as well as a collective pattern of migration (Toh et al., 2018). For multi-drug resistance assays, the microfluidic single-cell technique enables the study of drug efflux inhibition on a single MDA-MB-231 TNBC epithelial cell detached from the cell culture (Parekh et al., 2019). Based on the dielectric proprieties of non-metastatic BC cells compared with non-tumor breast epithelial cells, microfluidic devices are also able to sort MCF7 and MCF10A cells from a cell mixture (Ur Rehman, 2022).

It is well known that the heterogeneous cellular, soluble and physical components of the TME play a key role in BC behavior and treatment response (Li et al., 2021). Thus, the ECM, as a key component of the TME, acts as a 3D mechanical support for cancer cells, where the EMT occurs (Garre et al., 2022). Consequently, matrigel, synthetic matrixes, as well as patient-derived scaffolds (PDSs) are used to reproduce the tumor ECM in organ-on-chip systems (Garre et al., 2022). Thus, Garre et al. (2022) developed a PDS method based on decellularized primary BC repopulated with standard BC cell lines, such as MCF7, T47D, and MDA-MB-231, to analyse the BC cell behavior under TME influence (Garre et al., 2022).

BC cells settle within a tumoral niche of cancer-associated cells, such as cancer/tumor-associated macrophages, cancer/tumor associated fibroblasts, and cancer-associated adipocytes (Neagu et al., 2021), so microfluidic systems have been used to model this cancer cell-stromal crosstalk (Blyth et al., 2023). Evidence suggests that the tumor-immune microenvironment (TIME) biology is widely involved in BC pathological behavior (Tzoras et al., 2022). Chemokines and growth factors produced by tumor-associated cells promote cancer cells survival and proliferation (Rahman et al., 2020). The recruitment of immune cells to a tumor site can be demonstrated using multi-cellular-on-chip platforms as engineered TIME-on-chip systems that involve cancer cells, monocytes, macrophages, and endothelial cells as key players in the TME, embedded within a gelatin hydrogel or a 3D matrix, to analyze the contribution of macrophages and monocytes to tumor progression, while T-cells have been associated with a significant decreasing in migratory behavior of both BC cells and macrophages (Manoharan et al., 2024; Aung et al., 2020). Moreover, the vasculature can be also included in cancer-on-chip models to emphasize BC cells-vasculature interaction, intravasation and extravasation (Jouybar et al., 2024). Microfluidic platforms are able to analyze the endothelial cells–induced BC cell invasion into collagen type I-rich stroma that is sustained by pro-migratory factors secreted by endothelial cells that metabolically regulate BC invasion toward a microvesssel (Tan et al., 2023).

Tumor-associated macrophages (TAMs) are the most abundant immune cells in the TME that promote angiogenesis and cancer cell metastasis, inducing tumor stemness and immune system suppression, and regulating energy metabolism (Huang et al., 2022). Thus, Mi et al. (2019), using a 3D microfluidic tumor-macrophage system, showed that TAMs promoted invasion of MDA-MB-231 TNBC cells, which are able to maintain the specific phenotype of TAMs and, moreover, to promote the differentiation of human monocyte U937 cells into TAMs (Mi et al., 2019). Lugo-Cintrón et al. (2020), using an *in vitro* 3D microfluidic model of the breast TME reproducing a tumor mass invading the stroma, showed that in the presence of normal breast fibroblasts, a fibronectin-rich collagen matrix increases metastatic MDA-MB-231 TNBC cells migration, due to an overexpression of matrix metalloproteinases secreted by the normal fibroblasts (Lugo-Cintrón et al., 2020). In addition, MDA-MB-231 TNBC cells in co-culture with cancer-associated fibroblasts showed the largest migration distance (Lugo-Cintrón et al., 2020).

Mesenchymal stem/stromal cells (MSCs) are self-renewing multipotent cells, which travel from the bone marrow and from the adipose tissue, as adipose-derived stromal/stem cells (ASCs), to the TME of the primary site of the tumor and produce factors that sustain BC progression and chemoresistance (Augimeri et al., 2023). Several studies showed that the aggressive BC cells are able to engulf MSCs, resulting in hybrid cancer cell population that promotes BC metastasis, by generation of mesenchymal-like phenotypes, invasion, and stem cell traits (Augimeri et al., 2023; Chen et al., 2019). Thus, microfluidic cell pairing chips for investigating MSCs engulfment in BC cells have been engineered to demonstrate that primary breast carcinoma and distant metastases contain hybrid BC cell population that expressed EMT biomarkers, such as zinc finger e-box binding homeobox 1 (ZEB1) and smooth muscle actin (SMA) (Augimeri et al., 2023). Adipose-derived stromal/stem cells and mature adipocytes are known to play a significant role in BC pathophysiology (Hamel et al., 2024). Thus, Rahman et al. (2020) proposed a microfluidic device co-culturing MDA-MB-231 TNBC cells and adipose-derived stromal/stem cells to study the intracellular communication between these cell populations in the TME (Rahman et al., 2020). These authors showed that tumor cells exhibited enhanced growth, a more aggressive phenotype and an increased resistance to paclitaxel, a common chemotherapeutic used in BC patients (Rahman et al., 2020).

BC-on-chip systems are engineered to model and monitor the key events involved in BC pathophysiology and progression, such as uncontrollably growth of tumor cells, their interaction with stroma and ECM components, effects of hypoxia, interstitial fluid pressure, growth factor gradients, and neovasculature (Subia et al., 2021; Piotrowski-Daspit et al., 2016). The loss of E-cadherin protein, a central component of cell-cell adhesion junctions, is a hallmark of the EMT process (Kang and Massagué, 2004). The loss of E-cadherin is consistently observed at EMT sites in cancer, and its loss increases the invasiveness of the tumor cells (Kang and Massagué, 2004). Consequently, both EMT and angiogenesis are important for cancer metastasis and different *in vivo* animal and *in vitro* cell culture models have been used to study these processes, but differences in animal and human cells has proven to be an issue (Cho et al., 2021b). However, microfluidic models present similar systems to the human body and allow for multiple cell cultivation to emphasize intracellular communication (Cho et al., 2021b). Thus, Piotrowski-Daspit et al. (2016) used a 3D microfluidic culture model to obtain aggregates of MDA-MB-231 human BC cells embedded within a gel of type I collagen to demonstrate that elevated interstitial fluid pressure within the core of solid tumors increases the BC cells collective invasion from primary tumor via SNAIL, vimentin (VIM), and E-cadherin (CDH1), all of them associated with the EMT process (Piotrowski-Daspit et al., 2016). Choi et al. (2015) developed a micro-engineered pathophysiological model of early-stage BC as BC-on-a-membrane chip that enabled co-culture of MCF10-ductal carcinoma *in situ* (DCIS) spheroids with human mammary ductal epithelial cells (HMT-3522) and mammary fibroblasts embedded in a 3D ECM to mimic the tissue landscape of DCIS for assessing the efficacy and toxicity of paclitaxel (Choi et al., 2015).

Gioiella et al. (2016) proposed a microfluidic BC model-on-a-chip to replicate the activation of the tumor stroma reaction during the neoplastic invasion (Gioiella et al., 2016). This chip was designed with a stromal and a tumor compartment filled with 3D micro-tissues, kept in contact by a discontinuous wall separating stromal and tumor chambers. Thus, the stromal compartment contains normal fibroblasts, to mimic the normal stroma, or cancer-activated fibroblasts, to mimic the activated stroma, while the tumor compartment was injected with malignant MCF7 BC cells. These authors reported the phenotypic activation of the tumor stroma and matrix metalloproteinases overexpression, as well as collagen reorganization and the overexpression of ECM components, such as fibronectin and hyaluronic acid in the stromal compartment (Gioiella et al., 2016).

**FIGURE 1:**
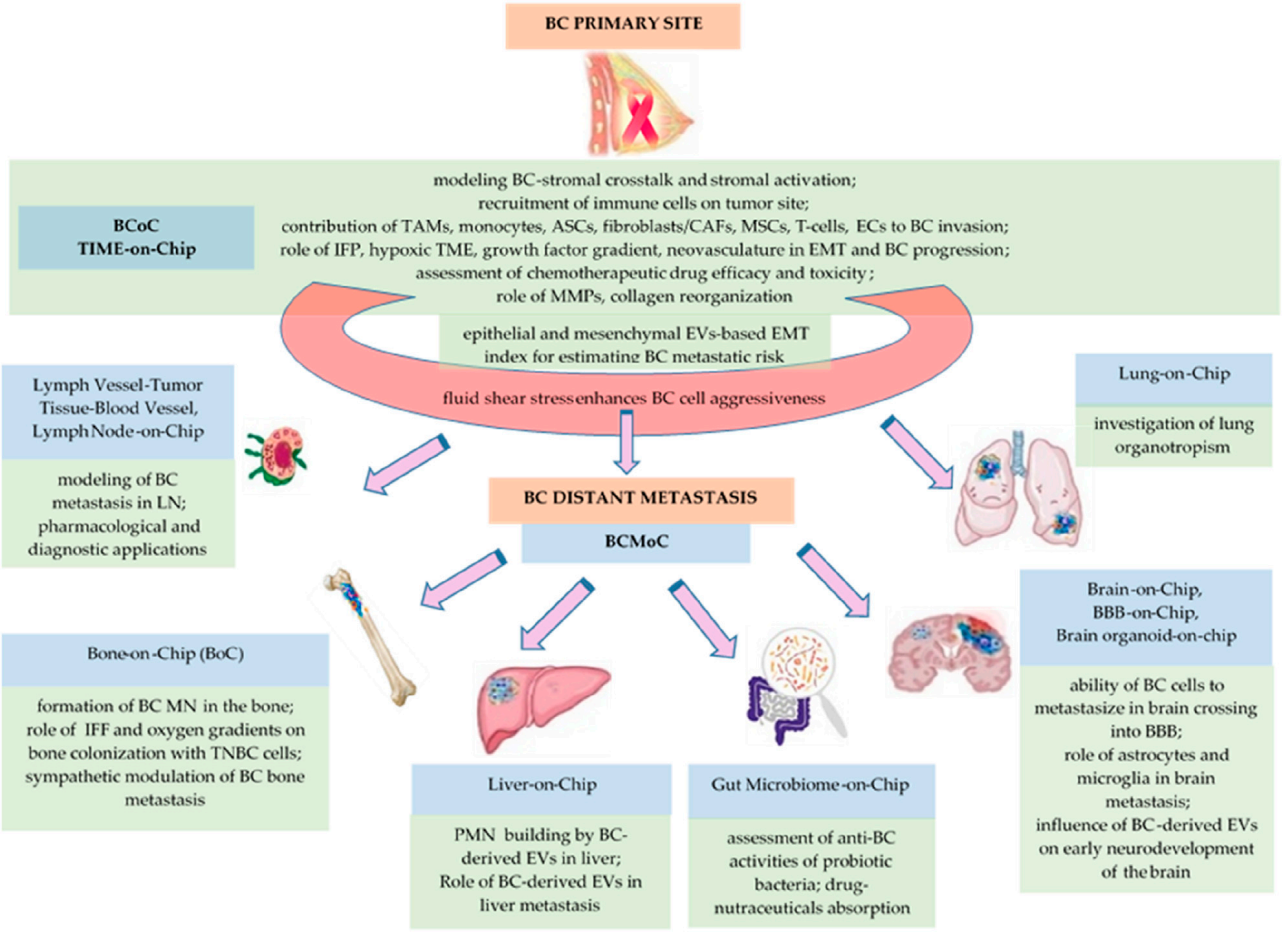
The biomedical relevance of BCoC and BCMoC systems for BCCC.

To analyse the effects of BC cells-monocyte interaction on T-cell recruitment, Aung et al. (2020) described a multicellular tumor-on-chip system involving cancer cells (MCF7 and MDA-MB-231 BC cell lines), monocytes (THP-1), T-cells (TALL-104), and endothelial cells (HUVECs) spatially distributed into a gelatine hydrogel (Aung et al., 2020). These authors concluded that the hypoxic environment in spheroid culture recruited more T-cells compared with dispersed cancer cells and the presence of monocytes synergistically increased T-cell recruitment in the tumor site, due to the differences in chemokine secretion that influence the endothelial permeability (Aung et al., 2020). Recently, Maulana et al. (2024) proposed a breast cancer-on-chip model integrating primary BC organoids to study patient-specific and safety testing of chimeric antigen receptor-T (CAR-T) cell efficacy, a type of genetically modified immunotherapy directed at cancer cells (Maulana et al., 2024). Thus, these authors developed a model with an integrated endothelial barrier that enables the transmigration of perfused immune cells, their infiltration into the tumor, and monitorization of cytokine relax (Maulana et al., 2024). The bio-medical relevance of these diverse BCoC platforms is presented in Table 1 and Figure 1.

**TABLE 1:** The bio-medical relevance of breast cancer-on-chip (BCoC) systems.

Chip role	Chip design	Chip relevance	References
simulation of BC cell migration and invasion across the basement membrane	3D microfluidic with MX1 BC cells for migration model	amoeboid- and mesenchymal-like motility and collective pattern of migration	Toh et al. (2018)
investigation of interstitial fluid pressure on the EMT	MDA-MB-231 BC cells embedded within a gel of type I collagen	high pressure increases BC cell collective invasion via EMT biomarkers (SNAIL, VIM, CDH1)	Piotrowski-Daspit et al. (2016)
early-stage BC modeling	MCF10-DCIS spheroids co-cultured with human mammary ductal epithelial cells and mammary fibroblasts embedded in a 3D ECM	assessment of efficacy and toxicity of paclitaxel	Choi et al. (2015)
investigation of BC-immune cell interactions to assess T-cell recruitment and role of TME on cytotoxic T-cell recruitment	CCs (MCF7, MDA-MB-231), monocytes (THP-1), ECs (HUVECs), T-cells (TALL-104)	hypoxic environment in spheroid culture recruited more T-cells compared with dispersed CCs; the presence of monocytes synergistically increased T-cell recruitment at the tumor site	Aung et al. (2020)
role of TAMs in BC invasion	3D microfluidic tumor-macrophage system with MDA-MB-231 TNBC cells	BC cells promote the differentiation of human monocyte U937 cells into TAMs	Mi et al. (2019)
role of breast fibroblasts and ECM components in modulation of BC cell migration	3D microfluidic model of TME including MDA-MB-231 TNBC cells	in the presence of normal human fibroblasts, a fibronectin-rich collagen matrix increases metastatic MDA-MB-231 TNBC cells migration due to an overexpression of MMPs	Lugo-Cintrón et al. (2020)
role of hybrid BC-mesenchymal stem/stromal cells to enhance chemoresistance and metastasis	cell engulfment-on-chip: MDA-MB-231 TNBC cell line and MSCs	primary BC and metastases contain hybrid BC cell population that express EMT biomarkers (ZEB1 and SMA) due to MSC engulfment in BC cells that stimulates metastasis	Augimeri et al. (2023), Chen et al. (2019)
evaluation of intercellular communication between BC cells and adipose-derived stromal/stem cells (ASCs)	microfluidic device co-culturing MDA-MB-231 cells and ASCs	tumor cells exhibit enhanced growth, a more aggressive phenotype, polarization toward ASCs, and an increased resistance to paclitaxel	Rahman et al. (2020)
replication of ECM activation during BC progression	tumor/inner chamber (MCF7); stromal/external chamber (3D tissue micro-modules formed by NFs or/and CAFs-assembled ECM)	MMPs overexpression in activated tissues; overexpression of fibronectin and hyaluronic acid, as well as collagen reorganization in the ECM during tumor progression	Gioiella et al. (2016)
evaluation of NPs/carbon dots-based drug delivery system	microvessel wall, ECM, BT549 (TNBC), T47D (non-TNBC) tumor spheroids, carbon dots functionalized with polyethylene glycol, acid folic, and doxorubicin	evaluation of dynamic transport behaviour and *in situ* cytotoxicity; rapid drug screening in pre-clinical studies	Chen et al. (2018)
multi-drug resistance assay	microfluidic single-cell technique	study of drug efflux inhibition on a single MDA-MB-231 TNBC cell	Parekh et al. (2019)
BC-on-chip	MDA-MB-231 TNBC cells aggregates embedded in a dextran-based hydrogel, CAR-T cell perfusion, and endothelial barrier	model for CAR-T cell infusion, recruitment, and infiltration into solid tumors for monitoring of dynamic cytokine release over more than 1 week	Maulana et al. (2024)

Abbreviations: ASCs, adipose-derived/stromal/stem cells; BC, breast cancer; CAFs, cancer associated fibroblasts; CAR-T, cells–chimeric antigen receptor-T, cell; CCs, cancer cells; CDH1 – E-cadherin gene; CDs, carbon dots; DCIS, ductal carcinoma *in situ*; ECM-extracellular microenvironment; ECs, endothelial cells; EMT, epithelial-mesenchymal transition; MMPs, matrix metalloproteinases; MSCs, mesenchymal stem/stromal cells; NFs, normal fibroblasts; NPs, nanoparticles; SMA, smooth muscle actin; VIM, vimentin; TAMs, tumor-associated macrophages; TME, tumor microenvironment; TNBC, triple negative breast cancer.

### 2.2 Breast cancer-liquid biopsy-on-chip (BCLBoC) systems recapitulate vascular spread of BC

Usually, liquid biopsy, a non-invasive technique to obtain tumor information from different patient’s body fluids (Shi et al., 2024), such as blood, urine, and saliva, includes circulating tumor cells (CTCs) and their derived exosomes containing tumor-derived microRNA (miRNA), mRNA, proteins and lipids, as well as cell-free tumor products, as circulating cell-free tumor DNA (cfDNA), which can capture the whole genetic profile of a tumor (Herzog et al., 2022). Consequently, different types of liquid biopsy allow for early stage cancer detection or for real-time monitoring tumor progression and cancer treatment (Mazzitelli et al., 2023). Thus, Gwak et al. (2019) designed a microfluidic device termed vortex-GMACS capable of on-chip cfDNA extracting from BC patient’s blood samples, reducing sample loss and processing time (Gwak et al., 2019). Thus, this chip assured an increase probability for binding cfDNAs to silica magnetic particles followed by cfDNA enrichment by capturing of these magnetic particles, in order to emphasize the mutational profile of tumor cell-derived DNA that could predict tumor progression (Gwak et al., 2019).

Small number of cancer cells, detaching from the primary tumor site or metastatic lesions, become epithelial, mesenchymal or mixed-types of CTCs, which can be detected in patient’s blood (Cai et al., 2023). CTCs are susceptible to enter EMT process, when CTCs progressively lose their epithelial characteristics and protein biomarkers and gain more mesenchymal phenotypic and molecular features (Cai et al., 2023). Consequently, CTCs intravasate and migrate into peripheral bloodstream, becoming responsible for cancer cell dissemination (Deng et al., 2022), so microfluidic systems play a significant role in detection and capture/isolation of these rare breast CTCs from human and murine blood samples for BC theranostics and prognostics (Macaraniag et al., 2023). Microfluidic-based CTCs enrichment platforms and downstream molecular analysis may represent a non-invasive alternative to invasive biopsy, or for patients whose breast tissue biopsies are unavailable (Cai et al., 2023). Cai et al. (2023) developed and validated a microfluidic-based platform for accurate CTCs isolation and downstream molecular analysis (Cai et al., 2023). This nanotube-CTC-chip, based on cytokeratins CK8/18, HER2, and epidermal growth factor receptor (EGFR), enables dynamic characterization of metastatic progression (Loeian et al., 2019). Abdulla et al. (2022) proposed an antibody labelled microfluidic chip for fast and accurately isolation of CTCs from BC patient’s whole blood by recognizing the transmembrane epithelial cell adhesion molecule (EpCAM) glycoprotein, the most commonly used biomarker for the capture and detection of CTCs, which is absent in blood cells (Abdulla et al., 2022; Sun et al., 2017). In addition, Zhang et al. (2021) proposed a label-free and size-based CTCs sorting microfluidic chip to isolate and identify many types of single CTCs, as well as CTCs clusters, from patient’s peripheral blood that may have a greater metastatic potential than single CTCs (Zhang et al., 2021).

Exosomes or small extracellular vesicles (EVs) contain tumor-derived proteins, nucleic acids, and lipids and are non-invasive biomarkers for cancer diagnosis and prognosis. Development of filter-electrochemical microfluidic chips allows for EVs isolation directly in patient’s whole blood based on surface protein detection (Wang et al., 2023). Thus, using MCF7, BT474, SK-BR-3, and MDA-MB-231 BC cell lines, specific tumor biomarkers of exosomes have been detected by Wang et al. (2023), such as prostate specific membrane antigen (PSMA), EGFR, cluster of differentiation 81/tetraspanin 1 (CD81/TAPA-1), and carcinoembryonic antigen (CEA) (Wang et al., 2023). Gwak et al. (2021) manufactured an integrated microfluidic chip, termed the HO-MOFF, for specifically separating epithelial and mesenchymal cell-derived EVs for calculation of the EMT-related index, which reflects the status of breast tumor and estimate the metastatic risk (Gwak et al., 2021). This chip selectively isolates EVs expressing the EpCAM epithelial biomarker and the CD49f mesenchymal biomarker from plasma samples of BC patients (Gwak et al., 2021). For rapid and efficient isolation of HER2+ SK-BR-3 BC cells, Parvin et al. (2023) integrated magnetic iron oxide nanoparticles functionalized with the anti-HER2 antibody with microfluidics (Parvin et al., 2023). Additionally, Mun B. et al. (2024) engineered a 3D-nanostructured microfluidic device arranged in a herringbone pattern nanochip functionalized with anti-human HER2 antibody for capture the exosomes derived from HER2+ positive BC cells in patient’s urine (Mun B. et al., 2024). A microfluidic chip based on three-segment hybridization and fluorescence imaging for multiple detection of miRNA biomarkers in BC was proposed by Gao et al. (2020) for early diagnosis of BC (Gao et al., 2020). In addition, Lim et al. (2022) proposed a chip, termed exosomal mRNA sensing microfluidic chip capable for detection of BC-derived exosomal mRNA in blood, using signal-amplifiable 3D nanostructured sensing hydrogels for detecting HER2 gene (Lim et al., 2022).

During migration for metastasis, cancer cells are exposed and differentially respond to fluid shear stress (Riehl et al., 2020) that can promote BC cell proliferation, invasive abilities, and chemoresistant phenotypes (Novak et al., 2019). A modular microfluidic system proposed by Ortega Quesada et al. (2024) emphasizes how the fluidic shear stress alters estrogen receptor phenotype in single and bulk MCF7 ER + BC cells (Ortega Quesada et al., 2024). These authors showed that the BC cells exposed to a high magnitude of fluidic shear stress (10 dyn/cm^2^) overexpressed the proliferation marker Ki67 as well as a higher phosphorylation level of proteins usually associated with a more aggressive phenotype in CTCs, such as p-AKT, p-mTOR, and p-STAT3, whereas no changes in p-ERK1/2 have been observed (Ortega Quesada et al., 2024). Concluding here, this microfluidic platform demonstrated that the BC cells from secondary metastatic sites can be more aggressive than primary BC cells (Ortega Quesada et al., 2024). The bio-medical relevance of diverse microfluidic and other BCLBoC is presented in Table 2 and Figure 1.

**TABLE 2:** The bio-medical relevance of breast cancer liquid biopsy-on-chip (BCLBoC) systems.

Chip type	Chip role	Chip design	Chip relevance	References
Vortex-GMACS-on-chip	cfDNA extraction from BC patient’s blood samples	microfluidic vortex along with a gradient magnetic-activated cfDNA sorter	analysis of DNA derived from tumor cells	Gwak et al. (2019)
Microfluidic chips	fast and accurately isolation of CTCs from BC patient’s whole blood	antibody labelled microfluidic chip	recognition of EpCAM in CTCs membrane	Abdulla et al. (2022)
	isolation of single and clustered CTCs from patient’s whole blood	label-free and size-based CTCs sorting	CTCs clusters may have a greater metastatic potential than single CTCs	Zhang et al. (2021)
	rapid and efficient isolation of HER2+ BC cells	magnetic iron oxide nanoparticles functionalized with the anti-HER2 antibody	isolation of HER2+ SKBR3 BC cell line	Parvin et al. (2023)
	separation of epithelial and mesenchymal cell-derived EVs	HO-MOFF: EMT-on-chip for EpCAM and CD49f biomarker detection in EVs	evaluation of status of BC and estimation of metastatic risk via EVs-based EMT-index in liquid biopsies	Gwak et al. (2021)
	capture of EVs derived from HER2+ positive BC cells in patient’s urine	nanochip arranged in herringbone pattern functionalized with anti-HER2 antibody	isolation of EVs from BC patient’s urine	Mun et al. (2024a)
	EVs isolation in patient’s whole blood	filter-electrochemical chip	multiple surface analysis of EVs; PSMA, EGFR, CD81, and CEA detected as tumor markers of EVs on MCF7, BT474, SKBR3, and MDA-MB-231 BC cell lines	Wang et al. (2023)
	multiple detection of miRNA biomarkers	chip based on three-segment hybridization	early detection of BC	Gao et al. (2020)
	detection of BC-derived exosomal mRNA in blood	exosomal mRNA sensing microfluidic chip	detection of *HER2*gene	Lim et al. (2022)
	role of fluid shear stress in induction of a more aggressive phenotype in metastatic cells compared to BC cell from primary tumor	modular microfluidic system	phenotype alteration in single and clustered MCF7 ER + BC cells; overexpression of Ki67 and phosphorylation of proteins associated with a more aggressive phenotype in CTCs (p-AKT, p-mTOR, and p-STAT3)	Ortega Quesada et al. (2024)

Abbreviations: BC, breast cancer; CEA, carcinoembryonic antigen; cfDNA, circulating cell-free tumor DNA; CTCs, circulating tumor cells; EGFR, epidermal growth factor receptor; EMT, epithelial-mesenchymal transition; EpCAM, epithelial cell adhesion molecule; EVs, extracellular vesicles; PSMA, prostate specific membrane antigen.

### 2.3 Breast cancer metastasis-on-chip (BCMoC) platforms recapitulate metastatic niche formation in distant organs

BC metastasis is a heterogeneous process, emphasizing organotropism for several end organs, so each site-specific metastasis has its unique cellular and proteomic landscape (Ali Moradi et al., 2023). Primary BC can spread to almost every place in human body, most frequently metastasizing to lymph nodes, bones (30%–60%), lungs (21%–32%), liver (15%–32%), and brain (4%–10%) (Firatligil-Yildirir et al., 2023; Liu C. et al., 2022), but the mechanisms of the BC subtype-specific organotropism is not yet very well understood (Wu et al., 2023).

It is known that bone recurrence occurs preferentially in luminal breast cancer (LBC) (Wu et al., 2023), HER2-positive tumors have the highest rates of metastasis to the liver (Rashid et al., 2021), while TNBC and basal-like subtype often develop metastases in brain that represents a challenge for cancer cells due to the blood-brain barrier (BBB) (Lee et al., 2015). Thus, microfluidic metastasis-on-a-chip biomimetic devices are required for successful explanation of the “seed and soil” hypothesis that sustains the interactions between CTCs and the organ specific microenvironment/relevant regulators in this particular niche.

Pre-metastatic niches (PMNs) formation is one of the key events in metastatic cascade that provides an appropriate environment for BC cells to colonize and progress into metastases (Li Y. et al., 2023). Platforms that mimics the PMNs can contribute to understand the complex interaction between tissue resident cells and metastatic tumor cells under the influence of the primary cancer (Ji et al., 2023). The interaction between BC cells and the TME from metastatic sites is known as a regulator of tumor progression (Kim et al., 2020). Moreover, at single-cell proteomics level, it is possible to integrate proteomics-based microfluidic chip and data-independent acquisition mass spectrometry (DIA-MS) to profile the proteomes of malignant cells (Gebreyesus et al., 2022). Thus, coupling organ-on-chip (OoC) models to MS is useful for analyzing the content of OoC with wide range of bio-medical applications (Hadavi et al., 2023). Thus, human lymph nodes (LN)-on chip includes biomimetic scaffold on which different cells can be seeded or grown, such as immune and stromal cells, and appropriate ECM components into dynamic flow conditions, taking also account about LN compartimentalization (Shanti et al., 2021). Normal and cancer cells can be loaded into biomimetic lymph node-on-chip systems that can be also connected to other organs-on-chip to study the relationship between lymph nodes and other organs (Shanti et al., 2021). Following the same principles, lung-on-chip, bone-on-chip, liver-on-chip, brain-on-chip, as well as gut microbiota-on-chip devices are really useful to deepen the understanding of the BCCCC and BCPCC. The bio-medical relevance of diverse BCMoC systems is presented in Table 3 and Figure 1.

**TABLE 3:** The bio-medical relevance of breast metastasis-on-chip (BCMoC) systems.

Chip type	Chip role	Chip design	Chip relevance	References
Lymph vessel-tumor tissue-blood vessel chip (LTB)	model for BC cells intravasation in lymph vessels, analysis of the intercellular communication in TME and role of inflammatory cytokines, stromal reaction, hypoxia, and nutrient deficiency during lymphatic metastasis	3-channels: HUVECs (mimic blood vessel wall), HLECs (mimic lymph vessel wall), hydrogel (ECM), injected with CTCs (MCF7, MDA-MB-231, SKBR3) pre-treated with TGF-β1 and IL-6 miRNAs	IL-6 exposure induces EMT and improved tissue invasion; the growth of HUVECs towards lymph vessel channel was observed by VEGF secretion from HLECs with IL-6 treatment	Cho et al. (2021a)
Lymph node-on-chip (LNoC)	model for BC metastasis in LN, pharmacological and diagnostic applications	3D collagen sponge (ECM), 4T1 BC spheroids (CCs), Jurkat cells (T-cells), BSA/TA NPs in the lymph flow	effect of drug carrier size/contrast agent on the penetration and accumulation of particles in spheroids modelling secondary tumor	German et al. (2023)
Liver-on-chip (liver-chip)	formation of PMN in liver; role of primary BC-derived EVs in breast cancer liver metastasis	endothelial and cancer compartment (LSECs, primary BC-derived EVs, BC cells (MCF7, MDA-MB-231))	activation of LSECs; EMT of BC cells; destruction of vascular barriers; upregulation of fibronectin in LSECs by TGFβ1; increased adhesion of CTCs to the liver microenvironment	Kim et al. (2020)
Bone-on-chip (BoC)	role of SNS signalling over bone metastatic BC	cancer compartment (bone tropic MDA-MB-231-BoM 1833 spheroids; neuron compartment (SH-SY5Y human sympathetic neurons); bone compartment (human peripheral blood derived osteoclasts seeded on top of a bone matrix)	dynamic interaction between BC cells, neurons, and osteoclasts; increased MMP1 and CTGF; bone tropic BC cells received synergistic inputs from neurons and osteoclasts, resulting in increased levels of pro-inflammatory cytokines	Conceição et al. (2022)
	formation of BC metastasis niche in bone	co-cultured *Murine calvaria* pre-osteoblasts (MC3T3-E1), MDA-MB-231^GFP^ BC cells	reorganization of the osteoblastic tissue; creation of large holes in surrounding matrix; long invadopodia from BC cells that protruded through matrix; CCs proliferate and organize into string of cells, parallel with elongated axis of the collagenous matrix	Hao et al. (2018)
	investigation of circulating neutrophils and breast CCs interactions during bone colonization	BC metastasis compartment: HUVECs^GFP^, BMSCs, fibroblasts, MDA-MB-231^RFP^) vascularized BME (ECs^GFP^, fibroblasts, BMSCs, O-BMSCs)) neutrophils	development of a perfusable vascularized BC metastasis into BME; CCs damage the microvascular network structure and permeability; metastatic-BME promotes neutrophil extravasation; neutrophils affect CCs viability in metastatic BME	Crippa et al. (2022)
Bone perivascular (BoPV) niche-on-a-chip with interstitial fluid flow	investigation of BC metastatic colonization in bone and drug resistance	hECs &BMSCs were co-cultured with MDA-MB-231 BC cells in 3D naïve bone matrix exposed to variable fluid flow velocities and oxygen gradients	formation of capillary-like structures in the niche; DTCs exhibited a slow-proliferative state and increased drug resistance in the BoPV niche-on-a-chip model	Marturano-Kruik et al. (2018)
Brain organoid-on-chip	BC cells derived-exosomes impair the early neurodevelopment of brain by BC metastatic transmission to placenta or fetus	hiPSCs-derived brain organoids, MCF7-derived exosomes	BC-derived exosomes affect the BB, CC, and MF of brain organoids; enhanced stemness of brain organoids under exosomes exposure; changes of forebrain development differentiation in brain organoids; increased stemness biomarker OCT4 and forebrain marker PAX6 for tumor progression and oncogenic features	Cui et al. (2022)
Blood-brain-niche-on-chip (BBN) and migration-on-chip	investigation of biological processes involved in breast cancer brain metastasis	flow chamber: MDA-MB-231 (TNBC)/JIMT1 (HER2+)/MDA-MB-231-BR/JIMT1-BR; brain niche chamber: brain microvascular ECs, astrocytes/microglia	astrocytes promote CCs extravasation through BBB; microglia influence CCs to remain in the proximity to the BBB; BBN secretions influence CCs migration and extravasation; cytokine response of the brain niche is influenced by interaction with MDA-MB-231-BR; astrocytic upregulated DKK1 influences CCs migration and extravasation across the BBB; CCs metabolism was rewired when stimulated with BN secretions; DKK1 overexpression led to overexpression of FGF13 and PLCB1 in CCs; extracellular DKK1 modulates CCs migration upon entering the brain niche	Westerhof et al. (2023)
Gut microbiome-on-chip (GMoC)	probiotic drug’s dosage screening	microfluidic device including *Lactobacillus acidophilus*, *L. casei*, *Bifidobacterium bifidum* strains and MCF7 BC cells	anti-BC activities of probiotic strains against BC cells	Salehi et al. (2023)

Abbreviations: BBB, blood-brain barrier; BMSCs, bone marrow-derived mesenchymal stem cells; BSA/TA NPs, bovine serum albumin/tannic acid nanoparticles; CCs, cancer cells; CTCs, circulating tumor cells; CTGF, connective tissue growth factor; DKK1 – Dickkopf Wnt signaling pathway inhibitor 1; DTCs, disseminated tumor cells; ECM, extracellular matrix; ECs, endothelial cells; EMT, epithelial-mesenchymal transition; EVs, extracellular vesicles; FGF-13, fibroblast growth factor 13; GFP, green fluorescent protein; HLECs, human lymphatic endothelial cells; HUVECs, human umbilical vein endothelial cells; IL-6, interleukin 6; LN, lymph node; LSECs, liver sinusoidal endothelial cells; MMP1 – matrix metalloproteinase 1; PLCB1 – phospholipase C beta 1; PMN, premetastatic niche; SNS, sympathetic nervous system; TGF-β1, transforming growth factor beta; TME, tumor microenvironment; TNBC, triple negative breast cancer; VEGF, vascular endothelial growth factor.

#### 2.3.1 Lymph vessel-tumor tissue-blood vessel chip (LTB) and lymph node-on-chip (LNoC)

BC metastasis involves the spread and proliferation of neoplastic cells into secondary sites via the blood and lymphatic circulation, the lymphatic system serving as the most significant route for dissociated BC cells dissemination (Cho et al., 2021b). BC cells migrate from the primary tumor to the sentinel lymph node via the efferent lymphatic vessels (Rahman and Mohammed, 2015). It is known that the early stages of metastasis are driven by the EMT and angiogenesis (Kang and Massagué, 2004). Cho et al. (2021a) developed a three-channel microfluidic chip, called LTB, to monitor lymphatic metastasis by mimicking the lymph vessel-tumor tissue-blood vessel compartments, emphasizing the inflammatory cytokine-mediated EMT and angiogenesis in BC, followed by intravasation of BC cells through the lymph vessels (Cho et al., 2021a). These authors used human umbilical vein endothelial cells (HUVECs), human lymphatic endothelial cells (HLECs), and hydrogel in between these two layers of endothelial cells to replicate the 3D-ECM from a natural tissue. This system was injected with: a) BC cells considered as CTCs (MDA-MB-231, MCF7, and SK-BR-3), pre-treated with two cytokines (transforming growth factor-β1 (TGF-β1), which regulates cell proliferation, migration, and differentiation (Hao et al., 2019), and inteleukin-6 (IL-6), which downregulates the expression of epithelial markers and upregulates the mesenchymal biomarkers in BC cells, activating the EMT process (Abaurrea et al., 2021)); b) oncogenic microRNAs for posttranscriptional regulation of pro-metastatic gene expression (Kim et al., 2018). Thus, the authors proposed a complex chip to analyse the intercellular communication in TME during the lymphatic metastasis, showing that IL-6 exposure to different subtypes of BC cells induced EMT and significantly improved tissue invasion (Cho et al., 2021a). Additionally, German et al. (2023) elaborated a complex microfluidic plug-and-play lymph node-on-chip (LNoC) for pharmacological and diagnostic applications, consisting of a 3D collagen sponge with a porosity comparable with the natural collagen ECM of the lymph node, 3D spheroids of 4T1 BC cell line that mimics secondary tumor in lymph node formed by metastasis process, injected Jurkat cells, an immortalized line of human T lymphocyte cells (Yang et al., 2019), and bovine serum albumin/tannic acid (BSA/TA) capsules, as drug delivery vehicles and contrast agents, in a flow rate that matched that of the lymph flow (German et al., 2023). In conclusion, lymph node-on-chip platforms can accurately reproduce the complex structure of lymph nodes, modelling immune and cancer cells motility, cell-cell interactions, influence of the dynamic mechanical stress, and drug distribution in BC research (Wang et al., 2024).

#### 2.3.2 Bone-on-chip (BoC)

Bone is the third most frequent site of cancer metastasis (Mansoorifar et al., 2021), so bone metastasis occurs at higher frequency in metastatic BC (70%) (Hao et al., 2018), due to disseminated tumor cells (DTCs), detectable in the bone marrow, that form bone metastases even after many years or decades of latency (Marturano-Kruik et al., 2018). Thus, cancer cells escaping blood or lymphatic vessels colonize the perivascular niches around blood capillaries in the bone marrow, where the oxygen, nutrients, and signalling factors pass from blood into the interstitial tissue, assuring the niche preservation (Marturano-Kruik et al., 2018). The complex interactions between metastatic BC cells and bone microenvironment include colonization, osteolytic destruction, and immunosuppressive bone environment (Pang et al., 2022). In the bone marrow compartment, the mean partial pressure of oxygen (PO_2_) ranges from 11.7 to 31.7 mmHg, with a mean of 20.4 mmHg (2.7%), indicating a hypoxic environment, so the construction of the hypoxic microenvironment based on bone-on-a-chip systems becomes a promising technology able to assure the oxygen gradients mimicking (Li et al., 2023c). To analyze the complex interaction between bone-resident cells and metastatic lung cancer cells under the influence of the primary cancer, Ji et al. (2023) fabricated a bone-on-chip (BoC) consisting of three compartments (dormancy niche, perivascular niche, and “vicious cycle” niche) that mimics the PMN formation (Ji et al., 2023). Hao et al. (2018) also reported a BoC for spontaneous growth of a 3D, mineralized, collagenous bone tissue co-cultured with MDA-MB-231 TNBC cells to study the BC cells interaction with bone matrix (Hao et al., 2018). Marturano-Kruik et al. (2018), to investigate BC metastatic colonization into bone and drug resistance, have also developed a microfluidic perivascular niche-on-a-chip, using a functional human triculture of endothelial cells (ECs), bone marrow-derived mesenchymal stem cells (BMSCs), and MDA-MB-231 TNBC cells, to imitate the metastatic colonization of a 3D bone matrix exposed to variable interstitial fluid flow velocities and oxygen gradients (Marturano-Kruik et al., 2018). Also, a phenotypical transition of BMSCs toward perivascular cell lineages that support the formation of capillary-like structures lining the vascular lumen has been reported (Marturano-Kruik et al., 2018).

A microfluidic model has been use to study of neutrophil-BC cell interaction in a BC metastasis to bone (Crippa et al., 2022). Thus, Crippa et al. (2022) introduced in this chip human red fluorescent protein (RFP)-transfected MDA-MB-231 TNBC cells within a bone-mimicking microenvironment containing BMSCs, fibroblasts, and green fluorescent protein (GFP)-transfected human umbilical vein endothelial cells (HUVEC^GFP^) that self-assambled into microvessel network and connected to bone-mimicking microenvironment with the metastatic BC seeds (Crippa et al., 2022). These authors observed that metastatic cells compromised the microvasculature structure, resulting in a decreased number of junctions and shorter network lenght, while the permeability was higher in the presence of cancer cells. Injected neutrophils in the microvascular network highly extravasated in the presence of cancer cells and increased the dying cancer cells (Crippa et al., 2022).

It is known that sympathetic neurons promote osteoblast differentiation through bone morphogenetic protein (BMP) signalling pathway, so these neurons are important for osteogenesis and bone remodeling (He et al., 2013). Therefore, Coceição et al. (2022) reported a human metastasis-on-chip model recapitulating neuro-BC interaction in a bone metastasis landscape, based on multicellular paracrine signalling between three compartments: sympathetic neurons (SH-SY5Y), bone tropic BC cells (MDA-MB-231-BoM 1833 spheroids), and human peripheral blood derived osteoclasts seeded on top of a bone matrix (Conceição et al., 2022). These authors showed that communications between sympathetic neurons and osteoclasts increased BC aggressiveness by overexpression of pro-inflammatory cytokines, such as IL6 and macrophage inflammatory protein 1α (MIP-1α), within the PMN (Conceição et al., 2022).

#### 2.3.3 Brain-on-Chip

10%–30% of all cancers metastasize to the brain and brain metastases are the most lethal cancer lesions (Oliver et al., 2020). After lung cancer, BC is the second most origin of brain metastasis (Watase et al., 2021), breast cancer brain metastasis (BCBM) accounting for 4%–10% of all secondary metastases from the breast (Firatligil-Yildirir et al., 2023). Treatment for BCBM has improved, but many struggle to cross the blood-brain barrier (BBB), making them ineffective for treatment (Westerhof et al., 2023). It is known that luminal-like BC is more prone to metastasize to the bone, whereas basal-like BC prefers soft tissues, such as the brain parenchyma and lung (Moccia and Haase, 2021), so 50% of BCBM have been attributed to the TNBC subtype (Terceiro et al., 2023). BBB can delay the passage of CTCs into the brain parenchyma, but the mechanisms by which BC cells cross the BBB still remains unclear (Terceiro et al., 2023). BBB is a highly specialized and selective barrier that includes the capillary endothelial cells (ECs) connected by adherent and tight junctions and surrounded by an endothelial basement membrane, pericytes, the parenchymal basement membrane, astrocyte foot processes covering 90% of the ECs surface, and microglia (Terceiro et al., 2023; Sigdel et al., 2021; Brosnan and Anders, 2018). In the presence of brain metastasis, the BBB can be disrupted and becomes much more permeable (Brosnan and Anders, 2018). Other cancers, such as lung cancer metastasis, have been studied using brain-on-chip microfluidic devices, which combines lung cancer cells and brain cells with a functional BBB (Firatligil-Yildirir et al., 2023). Brain-on-chip models can also be used to study how BC cells metastasize into the brain. BBB-on-chip can be engineered using astrocytes and bone marrow microvascular endothelial cells to mimic physical cell-cell interaction, vascular mechanical cues and cell migration of the BBB (Subia et al., 2021). The MDA-MB-231 TNBC cell line injected onto brain-on-chip demonstrated that that BC cells have the ability to cross into the BBB along with lung and melanoma cancer cells, whereas other cancerous cells cannot cross the BBB (Xu et al., 2016).

A study by Westerhof et al. (2023) used a blood-brain niche (BBN)-on-chip that recapitulated the BBB and niche to determine how metastatic BC cells affect the brain, mainly to characterize how astrocytes and microglia attract metastatic BC cells (Westerhof et al., 2023). Two BC cell lines were used, MDA-MB-231 (TNBC) and JIMT1 (HER2+), along with two brain metastatic niches (MDA-MB-231-BR and JIMT1-BR) (Westerhof et al., 2023). It was determined that the brain metastatic BC cell lines behaved differently than their parental counterparts in that the MDA-MB-231-BR cells crossed into the BBN via astrocytes and endothelial cells, whereas the JIMT1 and JIMT1-BR cells seemed to show minimal contact with the astrocytes and endothelial cells (Westerhof et al., 2023). Additionally, when exposed to the metastatic TNBC cells, the astrocytes had increased secretion of inflammatory cytokines, indicating their potential role in promoting metastasis, while also remodelling the metabolome of the BBN (Westerhof et al., 2023), therefore indicating that astrocytes are essential in promoting and progressing BCBM. Moreover, astrocytic Dickkopf Wnt signaling pathway inhibitor 1 (DKK1) was overexpressed and stimulated brain metastatic BC cell migration by increasing in gene expression of fibroblast growth factor 13 (FGF-13), known to be promote metastasis of the aggressive TNBC (Johnstone et al., 2020), and phospholipase C beta 1 (PLCB1), also upregulated in highly metastatic BC cells (Sengelaub et al., 2016).

Cui et al. (2022) used a brain organoid-on-chip with human induced pluripotent stem cells (hiPSCs)-derived brain organoids and tested the influences of MCF7-derived exosomes on early neurodevelopment of the brain and the tumorigenesis of brain organoids (Cui et al., 2022). Thus, in the presence of BC-derived exosomes, brain organoids overexpressed the octamer-binding transcription factor 4 (OCT4), known to maintain the stemness features in cancer stem cells (Zhang et al., 2020), as well as the paired box 6 protein (PAX6), which plays an important role in development of human neuro-ectodermal epithelial tissues and sustains cell proliferation in invasive ductal carcinoma of the breast (Xia et al., 2015).

#### 2.3.4 Lung-on-Chip

Lung is one of the most prevalent secondary metastatic site for BC cells, with an estimated 21%–32% of BC patients whose cancer has metastasized in the lungs (Firatligil-Yildirir et al., 2023). Zhu et al. (2023) showed that lung-on-chip models can be used to culture normal human alveolar epithelial cells, HUVECSs, respiratory muscle tissue cells, primary lung cancer cells or a single lung cancer cell line, lung cancer fibroblast lines, and other types of cellular components (Zhu et al., 2023). Thus, lung-on-chip platforms were able to investigate the cause of BC cells spread into the lung parenchyma and why triple-negative and HER2-enriched BC cells prefer to invade the lung sites (Firatligil-Yildirir et al., 2023). Other studies based on-a-chip containing an intact endothelial monolayer showed that metastatic BC cells are more prone to cross the endothelial barrier when in the presence of a stimulated lung microenvironment (Cauli et al., 2023). It has also been shown that the upregulation of different chemokine receptors has been related to metastasis. Thus, the upregulation of C-X-C motif chemokine ligand 12 (CXCL12) and chemokine receptor type 4 (CXC4) interactions with target ligands on the pre-metastatic niches of target lung tissues has been observed (Firatligil-Yildirir et al., 2023). Other studies are focusing on hypoxia induced lung cancer metastasis and screening of drugs (Liu X. et al., 2022), and how anticancer and immune cells are transported into the TME (Kim et al., 2022).

#### 2.3.5 Liver-on-Chip

Liver is the third most common metastatic site for BC (Liu C. et al., 2022), so the secondary or metastatic BC in the liver is a complex process associated with poor prognosis (Kim et al., 2020; Liu C. et al., 2022; Rashid et al., 2021). The interaction between BC cells and hepatic microenvironment in BC liver metastasis (BCLM) is crucial for development of effective treatments and prevention (Liu C. et al., 2022). For accomplish this purpose, Kim et al. (2020) proposed a microfluidic human liver-on-chip, termed the liver-chip, that recapitulates BCLM (Kim et al., 2020). This liver-chip recapitulates the formation of a premetastatic niche to emphasize the role of BC-derived EVs in liver metastasis. These authors demonstrated that BC-derived EVs activate liver sinusoidal endothelial cells (LSECs) in the liver-on-chip, inducing EMT and destruction of vessel barriers (Kim et al., 2020). Moreover, at proteomic level, the transforming growth factor β1 (TGFβ1) in BC-derived EVs overexpresses an adhesive ECM protein on LSECs, fibronectin, sustaining the adhesion of BC cells to the liver microenvironment.

#### 2.3.6 Gut microbiome-on-chip (GMoC)

The human gut is important for food digestion, absorption, disease regulation and immune function (Xiang et al., 2020). The function of the gut as a barrier aids in limiting the transport of compounds and microbes in and out of the digestive system, while also protecting the body from the passage of unwanted substances and the expansion of pathogenic organs (Ashammakhi et al., 2020). Gut organs-on-chip (GoC) models allow for the study of the absorption, metabolism and transport of drugs delivered orally (Ronaldson-Bouchard and Vunjak-Novakovic, 2018). Moreover, these models were designed to mimic a large surface area while also including symbiotic microbial flora (Ronaldson-Bouchard and Vunjak-Novakovic, 2018). Thus, GMoC models can be used to monitor drug absorption in the intestinal tract, interactions between host and intestinal microorganisms and nutritional metabolism (Xiang et al., 2020).

Generally, in cancers, the gut microbiome has been shown to play a significant role in tumor progression due to increased inflammation in the body (Mittal et al., 2023). A study on colorectal cells and the GoC model shows that the abundance of bacteria could promote the growth of cancer cells, as most bacteria will secrete lipopolysaccharides and peptidoglycans then can enter the gut and cause epigenetic reprogramming, which can lead to increased pro-inflammatory cytokine secretion, reduced clearance of pathogens and cancer cells and reduced intestinal barrier fortification (Mittal et al., 2023). Lee et al. (2024), using a GMoC including Caco-2 cells (µGut) mimicking intestinal structure and function and incorporating tumorigenic bacteria, analysed the µGut disruption and pro-tumorigenic signal activation (Lee et al., 2024). Beneficial gut microbe *Lactobacillus ssp*., as important human intestinal probiotics, added as a pre-treatment, prevented the pathogenic effect of pro-tumorigenic bacteria (Lee et al., 2024).

Bernardo et al. (2023) sustained a wide association between the microbiota composition in the digestive tract and BC progression, due to modulation of estrogen levels and immune response (Bernardo et al., 2023). For example, findings suggested that *Lactobacillus acidophilus* (*L. acidophilus*) can act as a putative immunostimulator, increasing the production of interferon gamma (IFN-γ) and decreasing interleukin 4 (IL-4) cytokine in the splenocytes of BALB/c mice bearing BC (Imani Fooladi et al., 2015). To emphasize the role of probiotic bacteria on BC progression, Salehi et al. (2023) used a microfluidic device to assess anti-BC activities of probiotic strains, such as *L. acidophilus*, *L. casei*, and *Bifidobacterium bifidum* against MCF7 cells that were concentration and time dependent (Salehi et al., 2023). Consequently, microfluidic devices can be used to simulate the effect of gut microbiome on cancer cell growth (Mittal et al., 2023).

## 3 BC-on-chip: understanding the pros and cons

Traditional 2D and 3D tissue culture-based systems (*in vitro*), animal models (*in vivo*), that overcome most of the limitation of these *in vitro* systems, as well as *ex vivo* BC models have certain limitations (Subia et al., 2021). Nevertheless, BC-on-chip *in vitro* platforms also work as available and promising tools for study of BC events. Many authors designed numerous types of microfluidics-based BC-on-chip systems, to recapitulate the 3D structure and function of primary tumor or metastatic niches at microscale level, reproducing as good as possible the dynamic condition that drives the metastatic cascade. These platforms perform many advantages: are non-invasive approaches, reduce animal testing by replacement of animal-derived products and transition to non-animal science, so can be less costly than animals, not present ethnical concern, accelerate translational and personalized medicine, allow for early BC detection, diagnosis, monitoring of BC growth and progression, BC modelling for chemotherapeutic drugs/nutraceuticals/nano-drugs delivery applications, many drugs and dosed of drugs being tested at the same time, discovery of new BC biomarkers into an automated, fast, and high-quality *in situ* manner at relatively low-cost, study the effects of host genetics and environmental factors on organ structure and physiology, assuring a high throughput sample screening at greater sensitivity and accuracy, and identification of new pathways in BC metastasis by the study the interactions between the stromal and breast tissue or multi-organ integration etc., (Subia et al., 2021; Palasantzas et al., 2023; Tian et al., 2022).

However, BC-on-chip models, as well as other cancer-on-chip platforms, still face some deep challenges, which need to be solved: fabrication is still complicated and requires advanced expertise and skills in nanotechnology, materials/nanomaterials, tissue engineering, and biomedicine-related technics to reproduce the native structure and functionality of organs and tumors; there are limitation and challenges concerning exploitation of quantitative data or for coupling of BC-on-chip systems and artificial intelligence for future BC management (Imparato et al., 2022; Moccia and Haase, 2021); serious limitations arise from impossibility to reproduce the breast gland in its integrality or to optimally exploit the biopsy samples volume and heterogeneity; BC cell lines cultured or co-cultured in these platforms are not optimal to reproduce BC intraheterogeneity, interheterogeneity and progression; creation of personalized BC-on-chip for accurate diagnosis, monitoring and drug testing is far to be a usual fact and to be applied in the clinic. It is known that the extracted biopsy represents a single snapshot at a time and may provide inadequate information about the tumor at a specific moment of its progression. Thus, inducible patient-derived stem cells (iPSCs), which are somatic cells that are reprogrammed to a pluripotent stage and possess the ability of differentiating into a wide spectrum of cell types, can be successfully used for multiple organs reconstruction for personalized medicine (Olgasi et al., 2020).

BC-on-chip platforms are not able to reproduce the specific, complex and dynamic biophysical and biochemical features of the tumor extracellular matrix that is constantly reshaped during BC progression. Fabrication of multiorgan-on-chip systems continues to be a challenge, due to the systemic nature of BC disease, impossibility to use the same culture medium to recapitulate the interstitial media of all distant inter-connected organs, to explain BC multi-organotropism. Extreme miniaturization also causes significant reorganization and changes in different organ proportions, leading to difficulties in engineering of appropriate vascular networks. Other challenges are related to real-time analysis for assessing cross-organ communication that perturbs the multi-organ-on-chip environment and affects the detection of low abundant molecules. Last but not least, adequate types of training in novel technologies, including different omics-based fields (proteomics, interactomics, transcriptomics, metabolomics, etc.) and advanced data analysis is required for holistic interpretation of large data resulting from these complex experiments. The most important challenges and limitation of BC-on-chip platforms are emphasized in Table 4.

**TABLE 4:** Limitations and challenges of BC-on-chip models.

Challenges	Limitations	Putative solutions	References
*General challenges*			
Fabrication of microfluidic devices is complicated	higher experimental costs, high costs of materials (glass, silicon, polymers, metals, quartz, ceramics), process time can be long and expensive, some physical proprieties of traditional materials are not desirable	use of cheaper and appropriate materials for cell analysis; creation of multifunctional and flexible stickers that can be combined depending on the experimental requirements; chip miniaturization; chip integration with other devices; use of 3D printing technology and 3D printable bioinks	Scott and Ali (2021), Niculescu et al. (2021), Cho et al. (2023)
Training the next-generation of highly specialized researchers and technicians to acquire specific skills in nanotechnology	professional background with high degree of interdisciplinarity/multidisciplinarity and transdisciplinarity (engineering, physics, chemistry, molecular and cell biology, stem cell research, clinical science, toxicology, pharmacology, imaging)	adequate types of training in novel technologies, including different omics-based fields (proteomics, interactomics, transcriptomics, metabolomics, etc.), data analysis	Moruzzi et al. (2023)
Tissue engineering techniques are complex	technical advances in biomaterials, stem cell engineering, microengineering, microfluidic technologies are necessary for reconstructing the tissue microarchitecture and dynamic spatio-temporal microenvironments, integration of biosensors to monitor tissue functionality and TME quality	recreate specific aspects of tissues for particular applications (drug transportation, monitoring barrier function, inflammatory response, EMT, angiogenesis, cancer cell intravasation and extravasation, study of cell-cell and cell-TME interactions)	Cho et al. (2023)
Holistic exploitation of quantitative data; coupling of BC-on-chip and artificial intelligence for future BC management	computational models are necessary to assess accuracy, robustness, reliability, reproducibility, efficiency, and relevance of standardized compounds, assays and biomarkers	exploitation of relationship between BC-on-chip and clinical data for translation of microfluidic platforms to the clinic; integration of multiomics data	Imparato et al. (2022), Moccia and Haase (2021)
Reproducing the breast gland on its integrality	each platform is designed to address a specific biological process and may not provide a complete network of biological processes that drive tumor behaviour	co-culture of many cell types of the breast or distant metastatic sites	Moccia and Haase (2021)
Sample volume and other features of the sample	tissue biopsies or explants (top-down chips) can be often too large to easily be incorporated into a chip	engineered (bottom-up chip) or natural miniature tissues are preferred, but require adequate natural matrices (Matrigel, collagen, hyaluronic acid, gelatine), or synthetic hydrogels (polyacrylamide, polyethylene glycol-fibrinogen, polylactic acid)	Picollet-D’hahan et al. (2021)
	cancer-on-chip no recreate tumor at real size (typically > 10^9^ cells)	increase the number and diversity of cells	Imparato et al. (2022)
	obtaining fresh tissue is challenging and use of patient sample in on-chip devices is limited	optimization of number, viability, growth of primary cells, limitation of variability in establishment and differentiation of iPSCs	Moccia and Haase (2021)
BC cell lines are not optimal for replicating tumor heterogeneity	lack of suitable BC cell lines for all BC subtypes, such as luminal-B cell line; BC cell lines accumulate mutations during subsequent series of cultivation	use of lesser studied and variable cell lines for all BC subtypes, other than MCF7, MBA-MD-231, and T47D	Moccia and Haase (2021), Li et al. (2023a), Dai et al. (2017)
Nanoparticle and therapeutic proteins delivery in the malignant BC cells and TME	study of therapeutic nanoparticles/proteins absorption, distribution, metabolism, elimination and toxicity	construction of platforms to predict nanoparticle behaviour with applications in nanmedicine; use of synthetic cells that synthesize therapeutic proteins on-demand inside tumors, killing malignant BC cells	Krinsky et al. (2018), de Roode et al. (2024)
Creation of personalized BC-on-chip for accurate diagnosis, monitoring, and drugs testing	genetic, physiologic, and biometric heterogeneity during BC progression sustains diversity of each individual; extracted biopsy represents a single snapshot at a time and the not represent the actual tumor heterogeneity and provide inadequate information	use of patient-specific iPSCs derived from somatic cells that are reprogrammed to a pluripotent stage, and differentiate into a wide spectrum of cell types	Palasantzas et al. (2023)
	primary cells can be inaccessible or they van not be isolated from tissues with high purity	patient-derived stem cells become more accessible with minimal invasiveness	Leung et al. (2022)
	primary cells often lose rapidly their tissue specific functions and viability *in vitro* and are not suitable for long-term studies	breast-specific cell type requires specific culture conditions	Leung et al. (2022), Moccia and Haase (2021)
Tumoral ECM is constantly reshaped during BC progression	reconstructing the dynamic TME of the primary BC (BC TME-on-chip); mammary tissue TME possesses specific, complex, dynamic biophysical and biochemical features that are difficult to recapitulate	animal and human-based model systems: BC biopsy or surgical resection, dissection, deconstruction, isolation of patient-specific stromal cells for reconstruction	Li et al. (2023a), Mun et al. (2024b), Liu et al. (2021)
		human-based model systems: repopulation of decellularized patient-derived scaffolds (PDSs) from fresh-frozen BC biopsies (including biobanked) with BC cell lines (MCF7, MDA-MB-231)	Leiva et al. (2023)
		inclusion of micro-devices able to control nutrients, growth factors, chemokine gradients and other parameters	Moccia and Haase (2021)
	difficulty in isolating specific cell subtype	established biomarkers for each subpopulation are required	Mun et al. (2024b)
	most models lack interactions with adipocytes and myoepithelial cells	inclusion of these cell types could enhance the quality of BC TME	Moccia and Haase (2021)
	addition of to many cell types affects maintenance of the chip	fabrication of standardized BC-on-chip models based on multiple co-cultured cells	Moccia and Haase (2021)
	reproduction of protein network from ECM	new matrix biomaterials with good biocompatibility are need to be synthetized; design novel tumor spheroids with inner biomimetic ECM easily penetrable by vascular network	Li et al. (2023a)
	incorporation of various types of immune cells on tumor-on-chip	improving technologies for long-term maintenance of the function of immune cells when immune cells are co-cultured with other cells for development of immunotherapies and tumor vaccines	Li et al. (2023a)
Fabrication of multi-organ-on-chip (some challenges are available also for single organ-on-chip)	BC is a systemic disease, so it is necessary to reproduce the dynamic interaction between BC-distant organs, to study BC cell spreading, seeding and metastasis and evaluate the efficacy and off-target toxicity of anti-BC therapeutics	coupling of potential metastatic niches, incorporation of physical, chemical, and molecular biosensors for multimodal and real-time detection of local factors, and online multiomics analysis	Picollet-D’hahan et al. (2021), Liu et al. (2021)
	culture media affect cell viability, phenotype, and senescence, so different interconnected organs require a microfluidic circuitry and specific perfusion parameters to reproduce *in vivo* situation; each breast-specific cell type and other organs specific cells require specific culture conditions	use of media to provide organ specificity, avoid infection risk, inter-sample variability, create patient-specific models; optimization of flow rate, prevention of dilution, assuring sufficient nutrients	Moccia and Haase (2021), Picollet-D’hahan et al. (2021), Mun et al. (2024b)
	use of serum leads of lack of reproducibility and standardization which is important for drug screen	use of appropriate media to provide organ specificity	Moccia and Haase (2021)
	scaling/extreme miniaturization can cause significant structural reorganization and changes in different organ proportions	use of scaling approaches (proportional, allometric, functional)	Picollet-D’hahan et al. (2021), Wikswo et al. (2013)
	engineering an appropriate vascular network	use of endothelial-tumor models instead tubing-based modeling; endothelium can be modelled using HUVECs, organ-specific microvasculature endothelial cells that are not always commercially available, endothelial cells isolated from biopsies or differentiated from iPSCs/mesenchymal stem cells	Picollet-D’hahan et al. (2021), Roudsari and West (2016)
	organs/cells must be exposed to biochemical, mechanical, and electrical stimuli for proper development and functioning	perfused medium must be supplemented with biochemical stimuli, such as sex hormones	Picollet-D’hahan et al. (2021)
		biophysical factors, such as generation of precise gradient fluid-flow shear stress, affect BC cell phenotype and aggressiveness leading to altered behaviour and increased aggressiveness at the metastatic site	Ortega Quesada et al. (2024)
	real-time and latter analysis for assessment of cross-organ communication can perturb the multi-organ-on-chip microenvironment and affect the detection of low-abundant molecules	minimal or no sample preparation, optimization of the amount of sample collected for molecular analysis	Picollet-D’hahan et al. (2021)
*Specific challenges*			
Liver-on-chip	induction of pluripotent stem cell-derived hepatocytes for personalized treatments adapted on individual patient’s needs; study of drug efficacy and toxicity	use of iPSC-based liver organoids that can differentiate into multiple hepatic cell types: more mature hepatocyte-like cells, cholangiocytes, stellate cells and Kupffer cells	Olgasi et al. (2020), Dalsbecker et al. (2022)
Lymph node-on-chip	simulating ECM and reproduction of the complex internal structure of LNs *in vitro*	constructing LN-on-chip with more complex structures to study the complex interaction of BC cells with stromal and immune cells	Wang et al. (2024)
Bone-on-chip	continuous monitoring of local factors that drive bone metastasis	integrating biosensors can provide non-invasive, continuous monitoring of the experiment progression	Zhang et al. (2023)
Brain-on-chip	functionality of brain is very complex and differ from person to person	correct assessment of the ratio of non-neuronal to neuronal cells depending on the brain region (1.2 astrocytes/neuron, 0.46 endothelial cells/neuron, 0.2 microglia/neuron), capillary density and blood flow, differences in structure of grey and white matter, shear stress at the endothelial barrier	Wikswo et al. (2013)
Gut microbiome-on-chip	complex fabrication process, small chip capacity, limited lifespan of cells, absence of supporting cells (microvascular endothelium, immune cells, goblet cells, muscle cells, enteric neurons), microvilli, and a mucus layer, possible contamination with impact on anti-BC drug/nutraceutical testing, lack of possibility to incorporate all important families of intestinal flora	use of biocompatible materials, incorporation of sensors, incorporating of iPSCs, developing collagen-based cilia-like structures, co-culturing complex microbial species in diverse media and oxygen condition, co-culture of aerobic host cells and anaerobic microbes	Thomas et al. (2023)

Abbreviations: ECM, extracellular matrix; HUVECs, human umbilical vein endothelial cells; iPSCs, inducible patient-derived stem cells; PDSs, patient derived scaffolds; TME, tumor microenvironment.

## 4 Conclusion

Breast cancer-on-chip, breast cancer liquid biopsy-on-chip, and breast cancer metastasis-on-chip models recapitulate and reproduce *in vitro* the central mechanisms involved in the Breast Cancer Continuum Concept (BCCC). These on-chip platforms reproduce BC hallmarks, recapitulating proliferation, TME/ECM, EMT/migration, intravasation, dissemination of CTCs through blood and lymphatic circulation, extravasation, distant tissues colonization and immune escape of cancer cells, reflecting the continuous flow of the metastatic cascade, by integrating multiple populations of BC cells and tumor-associated cells, which progress from the primary tumor site toward distant organotropic organs. Nevertheless, BC-on-chip *in vitro* platforms also work as available and promising tools for study of BC events. However, BC-on-chip models, as well as other cancer-on-chip platforms, still face some deep challenges, such as fabrication that remains still complicated and requires advanced expertise and skills in nanotechnology, materials/nanomaterials, tissue engineering, and biomedicine-related technics. Fabrication of personalized BC-on-chip for accurate diagnosis, monitoring and drug testing is far to be a usual fact and to be applied in the clinic. Fabrication of multiorgan-on-chip systems continues to be a challenge. Other challenges are related to real-time analysis for assessing cross-organ communication that perturbs the multi-organ-on-chip environment and affects the detection of low abundant molecules. Last but not least, adequate types of training in novel technologies, including different omics-based fields (proteomics/peptidomics, interactomics, transcriptomics, metabolomics, exposomics, pharmacogenomics, etc.) and accurate data analysis is required for a holistic interpretation of large data resulting from these complex experiments. Therefore, the aim of this review was to summarize and analyse the main bio-medical roles of tumor-on-chip platforms that can be used as tools to study the BCCC.

BC cell lines are often used as *in vitro* models to mimic *in vivo* systems for understanding of cancer development (Yu et al., 2017). Thus, the most frequently used BC cell lines to populate different BC-on-chip systems are MDA-MB-231, MCF7, T47D, BT549, MX1, 4T1, and SK-BR-3. MDA-MB-231 TNBC cell line is the most used model for the late-stage BC, usually associated with BC metastasis, frequently introduced into tumor-on-chip systems. MCF7 is a commonly used BC cell line used for estrogen receptor (ER)-positive BC cell experiments (Comşa et al., 2015). T47D cell line, as the MCF7 cell line, has several characteristics specific to the mammary epithelium, representing luminal A condition (ERα-positive, progesterone receptor (PR)-positive/negative, and HER2-negative) but is more susceptible to progesterone than the MCF7 cell line (Yu et al., 2017). BT549 is also a TNBC cell line characterised by high tumorigenicity and metastatic potential (Sun et al., 2022). SK-BR-3 is one of the most widely used BC cell line for model HER2-positive breast cancers (Nattestad et al., 2018). The 4T1 murine mammary cancer cell line is one of the most widely used BC models that shares important molecular characteristics with human TNBC, being a common model for metastatic tumors (Schrörs et al., 2020). Finally, human breast adenocarcinoma cell line MX1 is infrequently used as a basal BC cell line (Vincent et al., 2015). Patient-derived primary BC cells can be also used in organ-on-chip platforms to create patient-specific tumor models, mainly for identification of drug response patterns or effective drug combination for cancer therapy (Mou et al., 2022). Evidence suggests that microfluidic chip platforms also handle with patient-derived breast circulating tumor cells from liquid biopsies, enabling high capture rates and identification of personalized drug response in patients with advanced metastatic disease and very limited chemotherapeutic possibilities (Schwab et al., 2022).

In addition, multiple cell lines are co-cultured with BC cell lines to mimic the local condition from primary breast cancer lesions and premetastatic/metastatic niches from distant organs. Thus, human umbilical vein endothelial cells (HUVECs) are a suitable model to study blood vessel endothelial cell function and can be co-cultured with osteogenic cells to decipher their specific molecular communications in bone-on-chip platforms that recapitulate bone metastasis of BC (Kocherova et al., 2019). Human lymphatic endothelial cells isolated from lymph node (HLECs) are used to study the function of the endothelium of lymphatic vessels during the process of lymphatic intravasation (Gong et al., 2019). Both HUVECs and HLECs can be co-cultured within a lymph vessel-tumor tissue-blood vessel chip to model BC cell intravasation into lymph vessels and to study the role of local factors during lymph node metastasis (Cho et al., 2021a). Liver sinusoidal endothelial cells (LSECs) are introduced in liver-on-chip platforms to mimic sinusoidal capillary channels of the liver, establishing a barrier with important physiological and immunological functions (Shetty et al., 2018). The SH-SY5Y human neuroblastoma cell line is one of the most widely used cellular model that can proliferate continuously (Kaya et al., 2024), being co-cultured with human peripheral blood derived osteoclasts and BC cells to study the dynamic interaction between cancer cells, sympathetic neurons and osteoclasts within bone-on-chip platforms used for analysis of bone metastasis formation. Human leukemia monocytic cell line THP-1 that can be used to study monocyte/macrophage functions, T lymphoblast cell line TALL-104, the immortalized line of human T lymphocytes or Jurkat cells, CAR-T cells, and various strains of intestinal bacteria, such as *Lactobacillus* sp. and *Bifidobacterium* sp. can be used as organ-on-chip components to recapitulate the mechanisms involved in BC tumorigenesis and spreading in preferential distant organs.

Thus, BCoC platforms develop multiple bio-medical advantages:

• allow for seeding, growth and co-culture of multiple cell lines to emphasize the mechanisms of the intercellular communication involved in BC progression;
• mimic the cyto-histo-architecture of the breast for study the mechanisms leading to cancer onset under the influence of BC risk factors (risk-on-chip platforms);
• reproduce BC cell migration and invasion across collagen barrier that mimics the structure of the basement membrane and stromal compartment, analysing both mesenchymal- and amoeboid-like motolity, as well as collective pattern of BC cell migration;
• reproduce the structure of the extracellular matrix;
• model BC-stromal crosstalk ans stromal activation during the neoplastic invasion;
• tumor immune environment-on-chip systems demonstrate the role or recruitment of immune cells on the tumor site, emphasizing the contribution of macrophages/tumor-associated macrophages and monocytes, adipocytes/adipose-derived stem cells, fibroblasts/cancer-associated fibroblasts, mesenchymal stem/stromal cells, T-cells, and endothelial cell-induced BC invasion;
• study the effects of the hypoxic tumor microenvironment, interstitial fluid pressure (IFP), growth factors gradients and neovasculature contribution in BC progression;
• sort different types of BC cell lines from a cell mixture;
• mimic the tissue landscape of ductal carcinoma *in situ* and invasive ductal carcinoma for assessing the pharmacodynamics, efficacy and toxicity of chemotherapeutic drugs;
• model the mechanisms of drug efflux inhibition in multi-drug resistance assays;
• emphasize the function of metalloproteinases, fibronectin, hyaluronic acid and collagen reorganization for BC progression.

Liquid biopsy, a minimally- or non-invasive technique to obtain tumor information from different patient’s body fluids, allow for early-stage BC detection, or real-time monitoring tumor progression and treatment. Microfluidic systems play a significant role in detection and capture/isolation of rare breast circulating tumor cells from blood samples for BC theranostics and prognostics. Moreover, extracellular vesicles are important sources of biomolecular information about the tumoral landscape.

Thus, the BCLBoC systems are useful for:

• analysis of circulating cfDNA that accurately reflect the genomic instability of the primary BC;
• rapid and accurately isolation of CTCs from whole patient’s blood, using antibody-labelled or label-free microfluidic chips and size-based CTCs sorting;
• rapid and efficient isolation of HER2+ BC cells, as well as EVs-derived from HER2+ BC cells from BC patient’s urine;
• separation of epithelial and mesenchymal BC cell-derived EVs for evaluation of status of BC and estimation of metastatic risk through EVs-based EMT-index;
• proteomics-based analysis of EVs;
• detection of miRNAs biomarkers and BC-derived exosomal mRNA in patient’s blood for early BC detection;
• study of the influence of fluid shear stress on BC cell phenotype/proteomes to explain how BC cells from metastatic sites become more aggressive than BC cells from primary tumor.

BC metastasis is a heterogeneous process, emphasizing organotropism for several end organs, such as lung, bone, liver, and brain, so each site-specific metastasis has its unique cellular and proteomic landscape. Thus, platforms that mimics premetastatic and metastatic niches in different organs (BCMoC) can contribute to deepen the understanding of complex interaction between tissue resident cells and metastatic BC cells under the influence of the primary cancer. Moreover, coupling organ-on-chip (OoC) models to mass spectrometry (MS) is useful for analyzing the content of OoC with wide range of bio-medical applications. Thus, the lymph vessel-tumor tissue-blood vessel platform is able to monitor lymphatic metastasis, demonstrating the inflammatory cytokine-mediated EMT and angiogenesis in BC, followed by intravasation of BC cells through lymph vessels. Lymph node-on-chip platforms can be used for pharmacological and diagnostic applications. Bone-on-chip models reproduce the interaction between BC cells and bone matrix in different conditions of interstitial fluid velocities and various oxygen gradients, as well as communication between neutrophils and BC cells or sympathetic neurons and osteoclasts interaction in BC metastasis to bone. Brain-on-chip systems study the ability of BC cells to metastasize in the brain, crossing the blood-brain barrier. Additionally, blood-brain barrier-on-chip and brain niche-on-chip showed how astrocytes and microglia interact with BC cells, while brain organoid-on-chip study the influence of BC-derived EVs on early neurodevelopment of the brain. Lung-on-chip platforms investigate the preference of TNBC and HER2+ cells to spread into lung, while liver-on-chip platforms recapitulates the breast cancer liver metastasis, emphasizing the premetastatic niche formation as well as the role of BC-derived EVs in liver metastasis. Finally, the gut microbiome-on-chip (GMoC) demonstrate the role of probiotic bacteria on BC progression, simulating the effect of gut microbiome on BC cells growth.

Integration of on-chip platforms and proteomics-based specific approaches could offer important cues about the molecular profile of the metastatic cascade, alowing for novel biomarker discovery, especially based on non-invasive/minimally invasive liquid biopsies. Novel platforms integrating specific proteomic landscape of human milk, urine, and saliva might be useful for early and non-invasive BC detection. Moreover, multi-organ-on-chip systems integrating surgically extracted patient-derived BC cells and patient-derived scaffolds have a great potential to study BC at integrative level, due to the systemic nature of BC, for personalized and precision medicine.
